# Assessing risk of breast cancer in an ethnically South-East Asia population (results of a multiple ethnic groups study)

**DOI:** 10.1186/1471-2407-12-529

**Published:** 2012-11-19

**Authors:** Fei Gao, David Machin, Khuan-Yew Chow, Yu-Fan Sim, Stephen W Duffy, David B Matchar, Chien-Hui Goh, Kee-Seng Chia

**Affiliations:** 1 Division of Clinical Trials and Epidemiological Sciences, National Cancer Centre Singapore, 11 Hospital Drive, Singapore 169610; 2 National Heart Centre Singapore, 17 Third Hospital Drive Avenue, Singapore 168752; 3 Health Services & Systems Research, Duke-NUS Graduate Medical School, 8 College Road, Singapore, 169857; 4 Department of Medicine, Duke University Medical Center, 2400 Pratt Street, Durham, NC, 27705, USA; 5 Medical Statistics Unit, School of Health and Related Research, University of Sheffield, Regents Court, 30 Regent Street, Sheffield, S1 4DA, UK; 6 National Registry of Diseases Office, Health Promotion Board, Ministry of Health, Singapore, 168937; 7 Wolfson Institute of Preventive Medicine, Barts and The London School of Medicine and Dentistry, Queen Mary University of London, Charterhouse Square, London, EC1M 6BQ, UK; 8 Centre for Molecular Epidemiology, National University of Singapore, Singapore, 138671

## Abstract

**Background:**

Gail and others developed a model (GAIL) using age-at-menarche, age-at-birth of first live child, number of previous benign breast biopsy examinations, and number of first-degree-relatives with breast cancer as well as baseline age-specific breast cancer risks for predicting the 5-year risk of invasive breast cancer for Caucasian women. However, the validity of the model for projecting risk in South-East Asian women is uncertain. We evaluated GAIL and attempted to improve its performance for Singapore women of Chinese, Malay and Indian origins.

**Methods:**

Data from the Singapore Breast Screening Programme (SBSP) are used. Motivated by lower breast cancer incidence in many Asian countries, we utilised race-specific invasive breast cancer and other cause mortality rates for Singapore women to produce GAIL-SBSP. By using risk factor information from a nested case-control study within SBSP, alternative models incorporating fewer then additional risk factors were determined. Their accuracy was assessed by comparing the expected cases (*E*) with the observed (*O*) by the ratio (*E*/*O*) and 95% confidence interval (CI) and the respective concordance statistics estimated.

**Results:**

From 28,883 women, GAIL-SBSP predicted 241.83 cases during the 5-year follow-up while 241 were reported (*E*/*O*=1.00, CI=0.88 to 1.14). Except for women who had two or more first-degree-relatives with breast cancer, satisfactory prediction was present in almost all risk categories. This agreement was reflected in Chinese and Malay, but not in Indian women. We also found that a simplified model (S-GAIL-SBSP) including only age-at-menarche, age-at-birth of first live child and number of first-degree-relatives performed similarly with associated concordance statistics of 0.5997. Taking account of body mass index and parity did not improve the calibration of S-GAIL-SBSP.

**Conclusions:**

GAIL can be refined by using national race-specific invasive breast cancer rates and mortality rates for causes other than breast cancer. A revised model containing only three variables (S-GAIL-SBSP) provides a simpler approach for projecting absolute risk of invasive breast cancer in South-East Asia women. Nevertheless its role in counseling the individual women regarding their risk of breast cancer remains problematical and needs to be validated in independent data.

## Background

The best-known statistical model available for predicting an individual woman’s chance of developing breast cancer is that derived using information from regularly screened Caucasian women from the USA participating in the Breast Cancer Detection Demonstration Project (BCDDP) [1]. This model uses age-at-menarche, age-at-birth of first live child, number of previous benign breast biopsy examinations, and number of first-degree-relatives with breast cancer as well as baseline age-specific breast cancer risks, to provide a predicted probability of invasive or in situ breast cancer development. Subsequently, the baseline hazard was modified using invasive breast cancer rates from the National Cancer Institute’s Surveillance, Epidemiology and End Results (SEER) program from 1983-7 to obtain the model we term GAIL [2].

GAIL is well calibrated among Caucasian women who received annual screening [1-3]. Although derived from a particular group of Caucasian women, GAIL also permits projections for women with differing characteristics including those of other ethnic groups. But, because of the wide variation in international breast cancer rates and the risk factors associated with breast cancer, GAIL may not always perform well [4-7]. For example, Kaur *et al*[5] concluded that GAIL only applied to their subpopulation of women who had received screening mammograms and is not readily applicable to all American-Indian and Alaska-Native women. Similar conclusions were found for women from the Czech Republic [6] and Italy [7].

Because breast cancer rates are higher for Caucasian than African-American women over 40 years, and the reverse for younger women, Gail *et al*[8] amended GAIL to account for this racial difference using data from African-American women participating in the Women’s Contraceptive and Reproductive Experiences (CARE) Study. Further this modified model, termed CARE by Gail *et al*[8], is more parsimonious in that age-at-birth of first live child and its interaction with the number of affected first-degree-relatives are no longer included. CARE fits the Women’s Health Initiative Studies [8] data well with 350 cases observed and 323 expected but under predicts risk in African-American women with previous breast biopsy examinations.

Breast cancer rates are increasing throughout Asia and it is the leading cancer among Singaporean women [9], although the incidence rate is markedly lower than that for Caucasian women with a different etiology, particularly an earlier age-of-onset. It is also likely that only a small proportion of Asian women have received regular mammograms based on coverage of available screening programs. Thus, it is important to recognize limitations of the breast cancer prediction models when counseling women of different ethnic groups. The aim of this paper is to examine and modify models of 5-year invasive breast cancer risk in participants of the Singapore Breast Screening Programme (SBSP) to account specifically for women of Chinese, Malay and Indian origins.

## Methods

### Components of the Gail Models

To estimate the probability of invasive breast cancer in women using Gail models several components for the calculations have to be determined, the values of which depend on the specific women concerned. If, apart from those aged 0-19 years and greater than 85 years, the age range is divided into 15 equal divisions of 5 years, with the end of the age-group *j* indexed by *τ*_*j*_ (Table 1) then, for an individual at age *a*=*τ*_*j*-1_ within a particular relative risk, *r*_*ij*_, the probability of developing breast cancer by age *a*+5 is given, following Gail (1989, Equation 6) [1], by

(1) $$P\left(a,a+5,r_{\mathrm{ij}}\right)=\sum_{j}\frac{b_{j}r_{\mathrm{ij}}}{b_{j}r_{\mathrm{ij}}+c_{j}}\frac{S\left(\tau_{j-1}\right)}{S\left(a\right)}\frac{C\left(\tau_{j-1}\right)}{C\left(a\right)}\left[1-\exp\left\{-5\left(b_{j}r_{\mathrm{ij}}+c_{j}\right)\right\}\right]$$

where *i* refers to the binary split of the current age (agecat=0,1) at 50 years.

**Table 1:** **Age-specific breast cancer incidence rates per 100,000 women years, *B ***_***j ***_**and competing mortality rates per 100,000 women years, *c ***_***j ***_**established for Caucasian women in the USA when developing GAIL and the comparative values for Singapore as a whole, and for the three main ethnic groups used in risk calculation of GAIL-SBSP and modified GAIL-SBSP**

			**Caucasian women (1983–1987)**		**Singaporean (Overall)**		**Singaporean (Chinese)**		**Singaporean (Malay)**		**Singaporean (Indian)**	
Age	*J*	*τ*_*j*_	*B*_*j*_	*c*_*j*_	*B*_*j*_	*c*_*j*_	*B*_*j*_	*c*_*j*_	*B*_*j*_	*c*_*j*_	*B*_*j*_	*c*_*j*_
0 – 19	1	20	0	0	0.3	11.7	0.2	10.5	0.5	17.4	0.5	11.0
20 – 24	2	25	1	49.3	1.9	14.3	1.8	11.9	1.1	25.7	2.2	12.9
25 – 29	3	30	7.6	53.1	8.1	14.2	7.0	12.5	15.9	22.5	4.9	19.7
30 – 34	4	35	26.6	62.5	24.8	19.8	25.5	16.8	27.7	47.0	5.9	14.8
35 – 39	5	40	66.1	82.5	57.6	32.6	60.3	29.4	60.0	53.9	31.6	34.8
40 – 44	6	45	126.5	130.7	118.7	58.5	121.5	51.6	123.7	98.6	87.9	60.7
45 – 49	7	50	186.6	218.1	162.6	106.2	169.1	95.1	145.1	175.1	128.4	111.9
50 – 54	8	55	221.1	365.5	187.0	182.1	193.6	165.1	158.4	297.5	147.2	212.6
55 – 59	9	60	272.1	585.2	204.3	319.1	211.0	271.1	167.0	617.7	181.5	445.9
60 – 64	10	65	334.8	943.9	199.1	565.9	202.1	489.6	209.3	1093.0	157.6	853.3
65 – 69	11	70	392.3	1502.8	193.9	989.5	199.8	856.5	131.9	2040.3	219.5	1158.5
70 – 74	12	75	417.8	2383.9	166.5	1798.6	161.3	1624.3	199.1	3034.6	152.5	1932.2
75 – 79	13	80	443.9	3883.2	179.0	3285.3	180.9	3001.6	128.6	5678.6	197.2	3662.0
80 – 84	14	85	442.1	6682.8	189.8	5681.0	191.4	5388.2	188.4	8681.2	125.0	6093.8
>=85	15	90	410.9	14490.8	166.2	11425.5	164.5	11352.1	117.6	13323.5	375.0	11000.0

In a USA context, the important risk factors, and their category weightings, for the development of relative risk, *r*_*ij*_, include the current age, age-at-menarche (agemen), age-at-first-live-birth (ageflb) (nulliparous coded 2), number of first-degree-relatives with breast cancer (numrel), and number of previous benign breast biopsies (nbiops), presence of atypical hyperplasia (atypical) and interaction terms (ageflb×numrel and nbiops×agecat) [1,2].

In equation (1), *b*_*j*_=*B*_*j*_[1–*AR*(agecat)] is the baseline age-specific composite breast cancer rate for age-group *j*, *B*_*j*_ is the age-specific breast cancer incidence rate and *AR* is the attributable risk in the broader age category within which *j* falls. When developing GAIL, *AR* for the USA Caucasian population was found to be approximately constant in those less than 50 years at *AR*_−49_=0.4771, and for older women at *AR*_50+_=0.4736.

For an individual in risk group *i* of age *a*=*τ*_*j*-1_, the probability of remaining breast cancer free up to the age, *τ*_*j*_, is estimated by *S*(τ_*j*_)=*S*(τ_*j* − 1_)exp(−*b*_*j*_*r*_*ij*_Δ). In addition, the age-specific hazard *c*_*j*_ of dying of other causes is assumed to be the same for all subjects in the age-group *j*. The probability of *surviving* competing risks up to the end of the age-group *j*, *τ*_*j*_, is estimated by *C*(*τ*_*j*_)=*C*(*τ*_*j* − 1_)exp(−*c*_*j*_Δ), where *C*(0)=1.

The Fortran program BCPTCARE of the National Cancer Institute calculates equation (1), for given values *r*_*ij*_, by combining these with data providing information on *a*, *B*_*j*_ and *c*_*j*_.

### Data sources

#### SBSP

SBSP recruited 29,193 female permanent residents and citizens of Singapore, including 24,609 ethnically Chinese, 1,630 Malay and 1,434 Indian, from 01 October 1994 to 28 February 1997. Women were eligible with no previously diagnosed cancers (except non-melanoma of the skin), no mammography within the past year or biopsy within the last 6 months. Prior to mammography, all attendees completed a questionnaire including demographics; reproductive and family histories; smoking; and menopausal hormone therapy use [10,11].

Included in the risk evaluation are those who were disease-free (including 33 *in situ*) at the time of breast cancer screening. In order to focus on incident breast cancer, women were included only if they were followed-up to be alive without disease (5 *in situ*) for the next 3 years. As the prevalent breast cancers are not included, the study women have a lower absolute risk than the general female population [12]. Thus the 'clock' was started 3 years from the date of their negative screen and, amongst these women, those who developed invasive breast cancer in the following 4-8 year period are the designated cases. Any women with *in situ* disease who then developed an invasive cancer were considered as invasive in the year of this latter diagnosis. Women with unknown age-at-menarche or date-of-diagnosis were excluded.

The study was approved by the Singhealth Institutional Review Board (2008/468/B) and National Cancer Centre Institutional Review Board (NC08-041). As this was a large population based study, with full anonymity of all data, direct consent from the participants is waivered.

#### Nested case-control study

To study risk factors for breast cancer, a nested case-control study was conducted within SBSP in 2006 [13]. Women who were screened-positive (including those with *in situ* disease) or developed invasive breast cancer before 2006 were defined as case patients. Control subjects were selected from those who did not have a breast cancer diagnosis at the time of study. These were matched to cases by 5-year at age-at-entry groups and calendar year of entry into the SBSP program and ethnicity. Data from these women was used to build a model to project absolute invasive breast cancer risk.

#### Follow-up and breast cancer ascertainment

Breast cancer incidence and death status for SBSP participants were notified as either detected through SBSP or subsequently through record linkage with the Singapore Cancer Registry (SCR). All whose death status was not captured were assumed alive at 1 March 2009. SCR includes all cases of cancer occurring in citizens and permanent residents (population near 5 million) between 1968 and 2008. Annual invasive breast cancer cases and annual non-breast cancer deaths are obtained from SCR and annual population numbers from Singapore Resident Population report (2003-2007) [14]. These were used to calculate average race-specific estimates of *B*_*j*_, and *c*_*j*_ for the period 2003-2007 (Table 1, Singaporean).

### Statistical analyses

To estimate the probability of invasive breast cancer for a different population one can assume that all the components necessary are already contained in GAIL. That is, the regression coefficients, *β*, (Table 2, BCDDP), together with *B*_*j*_ and *c*_*j*_, remain as those specified when formulating that model (Table 1, Caucasian women). The calculated probabilities can be applied to an age-specific group of interest to provide the expected number of cases, *E*. This can then be compared to the actual number of cases observed, *O*. A ratio of *E*/*O* = 1 indicating perfect agreement within that age category. The corresponding 95% confidence interval (CI) is: $\frac{E}{O}\exp\left[\pm 1.96\times \sqrt{\frac{1}{O}}\right]$[2,3]. If *k* age categories are concerned then, under the null hypothesis, ∑(*O* – *E*)^2^/*E* follows a *χ*^2^ distribution with *k* degrees of freedom [8].

**Table 2:** **Breast cancer risk factors, and associated regression coefficients (*β*), used in developing the alternative Gail-based models**

**Risk factor**	**Categories**		**BCDDP**		**GAIL-SBSP**	**GAIL-SBSP (FULL)**	**S-GAIL-SBSP**	**E-GAIL-SBSP**
		**Code**	***β***	**Code**	***β***	***β *(CI)**	***β *(CI)**	***β *(CI)**
***Main effects***								
AGECAT (y)	<50	0	0.01081	0	0.01081	0.807	–	–
	≥50	1		1		(–0.73 to 2.35)		
AGEMEN (y)	≥14	0	0.09401	0	0.09401	0.240	0.238	0.204
	12–13	1		1		(0.05 to 0.43)	(0.05 to 0.43)	(0.01 to 0.39)
	<12	2		2				
AGEFLB (y)	<20	0	0.21863	0	0.21863	0.185	0.183	0.170
	20–24	1		1		(0.016 to 0.31)	(0.06 to 0.31)	(0.03 to 0.31)
	25–29 or nulliparous	2		2				
	≥30	3		3				
NUMREL	0	0	0.95830	0	0.95830	0.844	0.777	0.774
	1	1		1		(–0.17 to 1.86)	(0.30 to 1.26)	(0.29 to 1.26)
	≥2	2		2				
NBIOPS	0	0	0.52926	0	0.52926	−15.877	–	–
	1	1		1		(–16.28 to –15.47)		
	≥2	2		1				
ATYPICAL	No	0	0.57405*	99	–	–	–	–
	Yes	1		99				
BMI (kg/m^2^)	< 23.0	–	–	0	–	–	–	0.380
	23.0 – 27.4			1				(0.22 to 0.54)
	≥ 27.5			2				
PARITY	≥ 3	–	–	0	–	–	–	0.203
	1 – 2			1				(0.03 to 0.38)
	0			2				
***Interaction terms***								
NBIOPS x AGECAT			−0.28804		−0.28804	16.138	–	–
						(Not estimatable)		
AGEFLB x NUMREL			−0.19081		−0.19081	−0.059	–	–
						(–0.61 to 0.50)		

* Given by Novotny *et al* (2006, Table 2) [6].

In a preliminary investigation, we noted that applying GAIL unchanged to Singaporean women substantially overestimated the number of invasive breast cancer cases: (*O*=241, *E*=401.54, *E*/*O*=1.67). Consequently our study aim was modified, to one investigating features of the local population which might influence the ultimate predictions while preserving the Gail-based modelling approach.

Breast cancer rates and competing mortality rates are much lower for Singaporeans of all ethnic groups than Caucasians in those over the age of 30; particularly so in those > 70 years (Table 1, Singaporean). To take into account such differences, which may influence the expected value *E*[7,8,15], we formulated GAIL-SBSP using the regression coefficients and *AR* derived from the BCDDP cohort (Table 2) combined with the average race-specific estimates of *B*_*j*_, and *c*_*j*_ for the period 2003-2007 for Singaporean women (Table 1).

In validating GAIL-SBSP we were unable to classify SBSP participants with respect to either history of previous benign biopsy or atypical hyperplasia status. Consequently those ever having previous benign breast biopsies [11] were categorized as a single biopsy, and atypical hyperplasia was categorized as unknown.

As it is known that the etiological factors for breast cancer vary according to, for example, ethnicity and/or geographical location of the women, the risk factors concerned and/or their weightings in the established Gail model may require some modification. To explore this, we used subjects from the nested case-control study. Relative odds were obtained by use of multiple logistic regression with the same independent variables and coding as GAIL-SBSP (Table 2). A simplified model (S-GAIL-SBSP) with only three variables – age-at-menarche, age-at-birth of first live child and number of first-degree-relatives with breast cancer was identified. In contrast, in order to explore whether adding other risk factors could predict invasive breast cancer with improved accuracy, the extended model (E-GAIL-SBSP) added ethnicity (ethnicity), parity (parity), smoking (smoking), body mass index (bmi), use of hormonal replacement therapy (hrt), use of oral contraception (oc) and waist-to-hip ratio (whr). The body mass index was categorized as <23.0, 23.0–27.4 and ≥27.5 kg/m^2^ (coded as 0, 1 or 2) following the World Health Organization (WHO) guideline for Asian populations [16]. To avoid missing any potentially important predictors *P*<0.05 was used for statistical significance to select variables for multivariate modeling. Finally E-GAIL-SBSP was created by taking only those variables with prognostic significance into the model.

Model di**s**criminatory accuracy was measured by the age-specific concordance statistic, using a logistic regression model of breast cancer status on the estimated risks. Thus each model was assessed by use of the area under the receiver operating characteristics curve (AUC) created by computing sensitivity and specificity [17]. The CI was based on the standard normal approximation. The average of the age-specific concordances used weights proportional to the number of women in each age group [18]. The variance for the average age-specific concordance was the sum, over the age groups, of the weight squared multiplied by the estimated variance of the age-specific concordance estimate. Age-groups with no cases are excluded from the calculations.

## Results

### GAIL-SBSP

Of the 29,193 women in SBSP, 28,883 were available for the 5-year risk assessment. A total of 241 invasive cases were observed and these are categorized by ethnicity and age-group together with the numbers predicted by GAIL-SBSP (Table 3). In total GAIL-SBSP predicted 241.83 cases (*E*/*O*=1.00, CI=0.88 to 1.14) – suggesting good model calibration (goodness-of-fit, *P*=0.957). This satisfactory prediction was also seen within all age groups (goodness-of-fit, *P*=0.092). This agreement was reflected in Chinese and Malay, but not in the relatively few Indian women as 17 cases were observed and only 10.16 predicted (*E*/*O*=0.60, CI=0.37 to 0.96).

**Table 3:** **Comparison of the expected cases (*E*) of invasive breast cancer predicted by each respective model, to the observed cases (*O*) in the Singapore Breast Screening Programme (SBSP) cohort for each ethnic group**

**Age at entry, (y)**	**No. of women followed**			**GAIL-SBSP**			**S-GAIL-SBSP**			**E-GAIL-SBSP**	
		***O***	***E***	***E*/*O *(CI)**	***P***	***E***	***E/O *(CI)**	***P***	***E***	***E/O *(CI)**	***P***
**Overall**											
45–49	109	0	0.95	–	0.092	0.97	–	0.111	0.96	–	0.006
50–59	13,911	144	124.11	0.86 (0.73 to 1.01)		124.86	0.87 (0.74 to 1.02)		149.12	1.04 (0.88 to 1.22)	
60–69	14,642	95	115.38	1.21 (0.99 to 1.49)		114.57	1.21 (0.99 to 1.47)		137.70	1.45 (1.19 to 1.77)	
70–74	221	2	1.40	0.70 (0.18 to 2.80)		1.40	0.70 (0.17 to 2.80)		1.69	0.85 (0.21 to 3.38)	
Total	28,883	241	241.83	1.00 (0.88 to 1.14)	0.957	241.80	1.00 (0.88 to 1.14)	0.959	289.47	1.20 (1.06 to 1.36)	0.004
**Chinese**											
45–49	93	0	0.85	–	0.440	0.87	–	0.480	0.84	–	0.076
50–59	11,795	119	109.98	0.92 (0.77 to 1.11)		109.64	0.92 (0.77 to 1.10)		127.35	1.07 (0.89 to 1.28)	
60–69	12,256	87	99.85	1.15 (0.93 to 1.42)		98.22	1.13 (0.91 to 1.39)		115.12	1.32 (1.07 to 1.63)	
70–74	195	2	1.21	0.60 (0.15 to 2.42)		1.20	0.60 (0.15 to 2.40)		1.44	0.72 (0.18 to 2.88)	
Total	24,339	208	211.88	1.02 (0.89 to 1.17)	0.790	209.93	1.01 (0.88 to 1.16)	0.894	244.75	1.18 (1.03 to 1.35)	0.019
**Malay**											
45–49	7	0	0.05	–	0.652	0.06	–	0.591	0.05	–	0.209
50–59	711	4	4.91	1.23 (0.46 to 3.27)		5.23	1.31 (0.49 to 3.48)		7.27	1.82 (0.68 to 4.85)	
60–69	889	2	5.41	2.71 (0.68 to 10.82)		5.67	2.84 (0.71 to 11.34)		7.72	3.86 (0.97 to 15.43)	
70–74	12	0	0.08	–		0.08	–		0.11	–	
Total	1,619	6	10.46	1.74 (0.78 to 3.88)	0.168	11.04	1.84 (0.83 to 4.09)	0.130	15.15	2.53 (1.13 to 5.62)	0.019
**Indian**											
45–49	5	0	0.03	–	<0.001	0.03	–	<0.001	0.02	–	0.002
50–59	683	16	4.75	0.30 (0.18 to 0.48)		5.08	0.32 (0.19 to 0.52)		6.98	0.44 (0.27 to 0.71)	
60–69	727	1	5.34	5.34 (0.75 to 37.90)		5.56	5.56 (0.78 to 39.49)		7.58	7.58 (1.07 to 53.79)	
70–74	7	0	0.04	–		0.04	–		0.05	–	
Total	1,422	17	10.16	0.60 (0.37 to 0.96)	0.032	10.71	0.63 (0.39 to 1.01)	0.055	14.64	0.86 (0.54 to 1.38)	0.537

In general, predictions were good amongst the various risk categories (Table 4). However, among women who had two or more first-degree-relatives with breast cancer, the numbers were under predicted (*E*/*O*=0.18, CI=0.04 to 0.71) while for those with no history there was excellent calibration (*E*/*O*=1.02, CI=0.90 to 1.16).

**Table 4:** **Comparison of the expected cases (*E*) of invasive breast cancer predicted by each respective model, to the observed cases (*O*) in the Singapore Breast Screening Programme (SBSP) cohort by risk factor category**

**Risk factors**	**No. of women followed**			**GAIL-SBSP**			**S-GAIL-SBSP**			**E-GAIL-SBSP**	
		***O***	***E***	***E/O *(CI)**	***P***	***E***	***E/O *(CI)**	***P***	***E***	***E/O *(CI)**	***P***
AGEMEN (y)											
≥14	18,652	142	148.50	1.05 (0.89 to 1.23)	0.890	139.32	0.98 (0.83 to 1.16)	0.945	165.22	1.16 (0.99 to 1.37)	0.035
12–13	9,279	89	83.81	0.94 (0.77 to 1.16)		90.61	1.02 (0.83 to 1.25)		109.59	1.23 (1.00 to 1.52)	
<12	952	10	9.53	0.95 (0.51 to 1.77)		11.87	1.19 (0.64 to 2.21)		14.66	1.47 (0.79 to 2.73)	
NBIOPS											
0	27,176	218	222.80	1.02 (0.89 to 1.17)	0.629	226.43	1.04 (0.91 to 1.19)	0.129	271.38	1.24 (1.09 to 1.42)	0.003
1	1,707	23	19.04	0.83 (0.55 to 1.25)		15.37	0.67 (0.44 to 1.01)		18.09	0.79 (0.52 to 1.18)	
NUMREL											
0	28,143	225	229.50	1.02 (0.90 to 1.16)	0.045	227.71	1.01 (0.89 to 1.15)	0.109	272.98	1.21 (1.06 to 1.38)	0.004
1	729	14	11.98	0.86 (0.51 to 1.44)		13.67	0.98 (0.58 to 1.65)		16.01	1.14 (0.68 to 1.93)	
≥2	11	2	0.36	0.18 (0.04 to 0.71)		0.42	0.21 (0.05 to 0.83)		0.48	0.24 (0.06 to 0.97)	
AGEFLB (y)											
<20	4,952	26	29.89	1.15 (0.78 to 1.69)	0.900	31.58	1.21 (0.83 to 1.78)	0.750	39.77	1.53 (1.04 to 2.25)	0.038
20–24	10,840	77	80.61	1.05 (0.84 to 1.31)		81.02	1.05 (0.84 to 1.32)		94.41	1.23 (0.98 to 1.53)	
25–29 or nulliparous	9,843	97	93.48	0.96 (0.79 to 1.18)		92.61	0.95 (0.78 to 1.16)		112.39	1.16 (0.95 to 1.41)	
≥30	3,248	41	37.85	0.92 (0.68 to 1.25)		36.59	0.89 (0.66 to 1.21)		42.90	1.05 (0.77 to 1.42)	
BMI (kg/m^2^)											
<23.0	10,237	68	89.31	1.31 (1.04 to 1.67)	0.018	88.34	1.30 (1.02 to 1.65)	0.028	75.94	1.12 (0.88 to 1.42)	0.026
23.0–27.4	12,225	107	101.99	0.95 (0.79 to 1.15)		102.09	0.95 (0.79 to 1.15)		124.26	1.16 (0.96 to 1.40)	
≥27.5	6,411	66	50.45	0.76 (0.60 to 0.970		51.29	0.78 (0.61 to 0.99)		89.21	1.35 (1.06 to 1.72)	
Parity											
≥3	20,197	147	156.72	1.07 (0.91 to 1.25)	0.632	157.01	1.07 (0.91 to 1.26)	0.615	178.11	1.21 (1.03 to 1.42)	0.041
1–2	6,410	68	63.16	0.93 (0.73 to 1.18)		62.80	0.92 (0.73 to 1.17)		78.57	1.16 (0.91 to 1.47)	
0	2,276	26	21.95	0.84 (0.57 to 1.24)		21.99	0.85 (0.58 to 1.24)		32.78	1.26 (0.86 to 1.85)	

### S-GAIL-SBSP

To estimate the relative risk function, we analyzed 439 invasive breast cancer cases (121 diagnosed at screening and 318 subsequently) and 1,198 controls from the nested case-control study (Table 5). As far as possible, those risk factors identified for GAIL were initially used to estimate the regression coefficients which were reported in Table 2 (GAIL-SBSP (FULL)). Using the same model structure there are some substantial differences, and a good deal of instability when estimating the interaction terms, as compared to those derived for GAIL. As a consequence, the simplified model S-GAIL-SBSP including only age-at-menarche, age-at-birth of first live child and number of first-degree-relative with breast cancer to obtain the relative risks (*RR*) was derived (Table 2, S-GAIL-SBSP). The corresponding *RR*s for each of the risk categories are given in Table 6 where they are compared with those used in GAIL (Table 6, BCDDP). Omitting age and number of previous benign breast biopsies and the interactions did not degrade the fit of the model (*P*=0.359).

**Table 5:** Baseline characteristics and odds ratios (OR) of invasive breast cancer in the nested case-control study

**Characteristics**	**Categories**	**Cases (N=439)**	**Controls (N=1,198)**	**Adjusted OR (CI)**	***P***
AGECAT (y)	< 50	2	11	1.00 (referent)	0.317
	≥ 50	437	1187	2.19 (0.47 to 10.13)	
AGEMEN (y)	≥ 14	246	772	1.00 (referent)	0.039
	12 – 13	173	377	1.23 (1.01 to 1.49)	
	< 12	20	49	1.51 (1.24 to 1.83)	
AGEFLB (y)	< 20	56	187	1.00 (referent)	0.015
	20 – 24	132	440	1.19 (1.04 to 1.37)	
	25 – 29 or nulliparous	185	425	1.42 (1.23 to 1.64)	
	≥ 30	66	144	1.69 (1.47 to 1.95)	
NUMREL	0	411	1162	1.00 (referent)	0.002
	1	25	36	2.15 (1.31 to 3.52)	
	≥ 2	3	0	4.62 (2.82 to 7.56)	
NBIOPS	0	398	1119	1.00 (referent)	0.234
	≥ 1	41	79	1.28 (0.85 to 1.94)	
ETHNICITY	Chinese	377	1051	1.00 (referent)	
	Malay	15	29	0.96 (0.58 to 1.59)	0.868
	Indian	25	65	1.30 (0.67 to 2.50)	0.434
	Others	22	53	1.02 (0.60 to 1.74)	0.936
BMI (kg/m^2^)	< 23.0	126	476	1.00 (referent)	<0.001
	23.0 – 27.4	207	497	1.43 (1.22 to 1.68)	
	≥ 27.5	106	223	2.04 (1.73 to 2.39)	
PARITY	≥ 3	263	801	1.00 (referent)	0.031
	1 – 2	112	276	1.22 (1.02 to 1.46)	
	0	64	121	1.49 (1.24 to 1.79)	
OC	No	278	743	1.00 (referent)	0.894
	Yes	161	455	1.02 (0.80 to 1.29)	
HRT	No	364	1022	1.00 (referent)	0.265
	Yes	75	173	1.19 (0.88 to 1.62)	
SMOKING	No	415	1126	1.00 (referent)	0.831
	Yes	24	72	1.05 (0.98 to 1.72)	
WHR	≤ 0.85	293	863	1.00 (referent)	0.074
	> 0.85	146	335	1.25 (0.98 to 1.60)	

**Table 6:** **Prevalence of breast cancer risk factors in the nested case-control study within Singapore Breast Screening Programme (SBSP) cohort and relative risks (*RR*) from SBSP and the Breast Cancer Detection and Demonstration Project (BCDDP)**

		**Cases**	**Control**	**Total (%)**		***RR***	
					**E-GAIL-SBSP**	**S-GAIL-SBSP**	**BCDDP**
**Women (*n*)**		439	1,198	1,637	1,637	1,637	5,998
AGEMEN (y)							
≥14		246	772	1,018 (62.19)	1	1	1
12–13		173	377	550 (33.60)	1.23	1.27	1.10
<12		20	49	69 (4.22)	1.50	1.61	1.21
AGECAT (y)	NBIOPS						
<50	0	2	10	12 (0.73)	–	–	1
	1	0	1	1 (0.06)	–	–	1.70
	2	–	–	–	–	–	2.88
≥50	0	396	1,109	1,505 (91.94)	–	–	1
	1	41	78	119 (7.27)	–	–	1.27
	2	–	–	–	–	–	1.62
AGEFLB (y)	NUMREL						
<20	0	54	182	236 (14.42)	1	1	1
	1	2	5	7 (0.43)	2.17	2.17	2.61
	≥2	–	–	–	4.70	4.73	6.80
20–24	0	123	431	554 (33.84)	1.19	1.20	1.24
	1	7	11	18 (1.10)	2.57	2.61	2.68
	≥2	2	0	2 (0.12)	5.57	5.68	5.78
25–29 or nulliparous	0	172	410	582 (35.55)	1.40	1.44	1.55
	1	13	15	28 (1.71)	3.05	3.14	2.76
	≥2	–	–	–	6.60	6.83	4.91
≥30	0	62	139	201 (12.28)	1.66	1.73	1.93
	1	3	5	8 (0.49)	3.61	3.77	2.83
	≥2	1	0	1 (0.06)	7.82	8.20	4.17
BMI (kg/m^2^)							
< 23.0		126	476	602 (36.82)	1	–	–
23.0 – 27.4		207	497	704 (43.06)	1.46	–	–
≥ 27.5		106	223	329 (20.12)	2.14	–	–
PARITY							
≥3		263	801	1,064 (65.00)	1	–	–
1–2		112	276	388 (23.70)	1.23	–	–
0		64	121	185 (11.30)	1.50	–	–

The differences in the *RR*s between the nested case-control study and BCDDP are largest only in the groups where the number of first-degree-relatives with breast cancer is two or more.

The simplified model with only three variables – age-at-menarche, age-at-birth of first live child and number of first-degree-relative with breast cancer utilized the modified *RR*s and predicted 241.80 cases (*E*/*O*=1.00, CI=0.88 to 1.14) (Table 3). The satisfactory prediction was seen in all ethnic groups although among Indian women, as was the case for GAIL-SBSP, the calibration was not entirely consistent across all age-groups.

Again similar to GAIL-SBSP, S-GAIL-SBSP predictions were relatively close amongst the various risk categories (Table 4). However, the model underestimated the observed incidence of breast cancer for women who had a biopsy although this was not statistically significant (*E*/*O*=0.67, CI=0.44 to 1.01) while for those without a biopsy there was a very good calibration (*E*/*O*=1.04, CI=0.91 to 1.19).

### E-GAIL-SBSP

To determine whether other risk factors could improve S-GAIL-SBSP performance, the effects of the Gail model risk factors were re-estimated in a multiple logistic regression that used subjects from the nested case-control study. We expanded the model by including ethnicity, parity, smoking, bmi, use of hormonal replacement therapy, use of oral contraception and waist-to-hip ratio to estimate the regression coefficients (Table 5). In addition to age-at-menarche, age-at-birth of first live child and number of first-degree-relatives with breast cancer, both parity and bmi were significantly associated with the probability of invasive breast cancer.

Following Gail *et al*[8], the *ARs* necessary to convert Singaporean age-specific invasive breast cancer rates to baseline rates for SBSP women were calculated. In order to match the follow-up period of the SBSP participants, this was based on invasive cases diagnosed over 1993-2002 [9,19] and over 2003-2007 [SCR unpublished]. Estimates of *AR* were 0.5356 for those younger than 50 years and 0.5397 for older women. These *AR*s and modified *RR*s were used to re-evaluate equation (1) and hence formulated E-GAIL-SBSP together with Singapore race-specific estimates of *B*_*j*_ and *c*_*j*_ (Table 1).

Overall, E-GAIL-SBSP predicted 289.47 cases (*E*/*O*=1.20; CI=1.06 to 1.36) and so was not able to satisfactorily capture the number of cases among the various risk categories (goodness-of-fit, *P*=0.004). Moreover, E-GAIL-SBSP statistically significantly underestimated the number of cases among women with two or more first-degree-relatives with breast cancer (*E*/*O*=0.24, CI=0.06 to 0.97).

### Comparison between the three models

**Figure 1:**
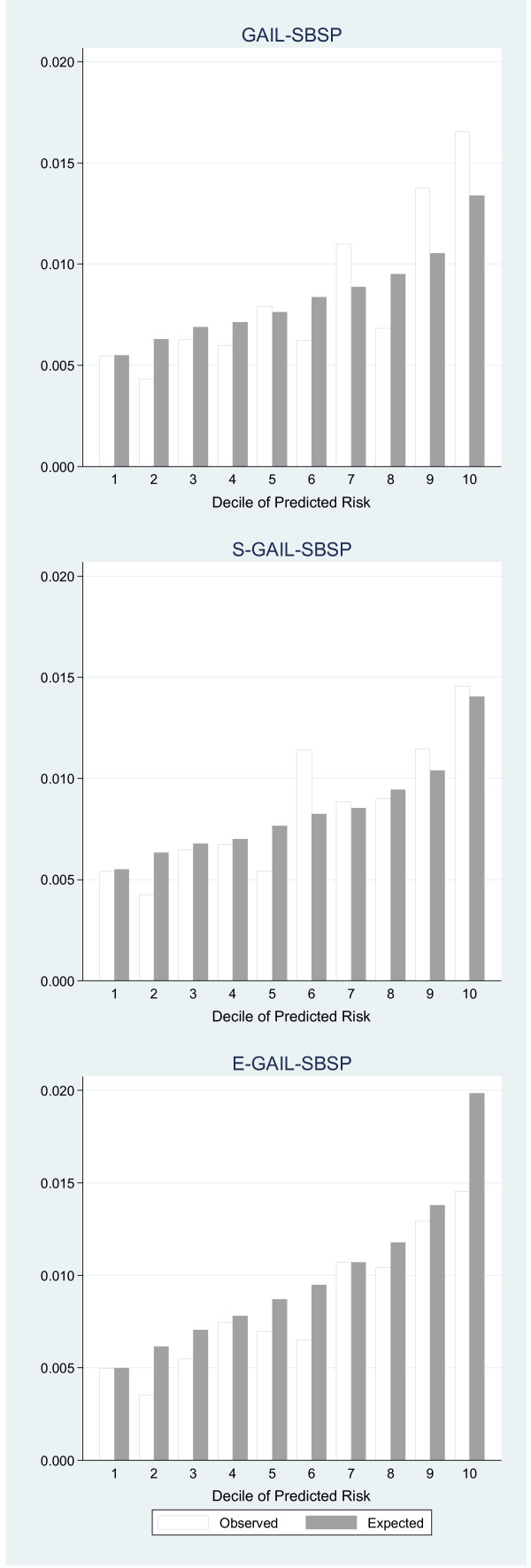
**Comparison of observed breast cancer risk with that predicted by each model.** The horizontal axis shows the grouping by deciles of risk and the vertical axis the observed and corresponding predicted risk.

In the calibration, the SBSP participants were divided into deciles of 5-year invasive breast cancer risks predicted by GAIL-SBSP, S-GAIL-SBSP and E-GAIL-SBSP, respectively. These predicted rates were compared with those observed in Figure 1. Thus, for example, GAIL-SBSP under predicted in the seventh, ninth and tenth deciles, there were generally closer predictions with S-GAIL-SBSP except in the sixth decile, and a considerable over prediction in the tenth decile with E-GAIL-SBSP. Clearly the addition of bmi and parity to E-GAIL-SBSP did not materially improve calibration.

**Figure 2:**
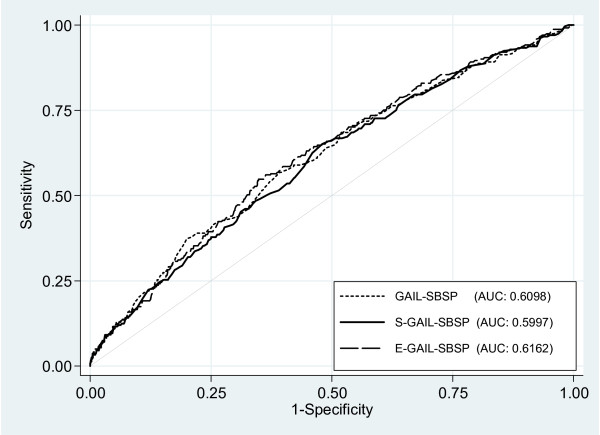
Receiver operating characteristic and corresponding area-under-the-curve (AUC) for the breast cancer risks predicted by GAIL-SBSP, S-GAIL-SBSP and E-GAIL-SBSP, respectively.

The unweighted average concordance statistics were very similar, and not statistically significantly different, with AUC = 0.6098 (CI=0.57 to 0.65), 0.5997 (CI=0.56 to 0.64), and 0.6162 (CI=0.58 to 0.65) for GAIL-SBSP, S-GAIL-SBSP and E-GAIL-SBSP, respectively (Figure 2). In addition, the estimated age-specific AUC of S-GAIL-SBSP for the intervals from 50 to 74 years were modest except in the oldest group; specifically, 0.5766 (CI=0.53 to 0.62) for those aged 50 **-** 59, 0.5838 (CI=0.53 to 0.64) for 60 **-** 69 years, and 0.8938 (CI=0.70 to 1.00) for 70 **-** 74 years.

## Discussion

Although first developed by MH Gail and his associates [1] some 25 years ago, the GAIL model continues to play an important role in predicting the 5-year risk of invasive breast cancer. Thus Schonfeld *et al*[15] have shown that GAIL remains well calibrated in more recent cohorts. However, although refinements have been made, application of the underlying methodology to non-Caucasian women has been limited and suggests that further modification may need to be made for its use in women of other ethnic groups. Singapore has a population which is predominantly of Chinese ethnicity but also with those of Malay and Indian descent and has also completed a large mammographic screening study involving 29,193 randomly selected women follow-up from which enables invasive breast cancer rates to be determined. Thus the very different breast cancer rates, and etiological risk factors varying in their presence and magnitude when compared to other populations, enables the GAIL model itself to be tested and variants (if relevant) to be established.

Retaining the GAIL model structure, but applying Singapore national and race-specific invasive breast cancer and other cause mortality rates, to develop GAIL-SBSP resulted in absolute risk projections that worked reasonably well as assessed by the comparison of observed and expected cases across all age groups and amongst the majority of risk categories (Tables 3 &4). In total 241 cases were recorded while GAIL-SBSP predicted 241.83 (*E*/*O*=1.00, CI=0.88 to 1.14). However, the model under predicted for the very few women who had two or more first-degree-relatives with breast cancer. Prediction was satisfactory for Chinese women but over-predicted for the Malay and Indian women. Since these latter groups each comprise of only 5% of the population studied, the accuracy of these specific predictions requires further investigation in residents of the Malaysian Peninsula and the Indian Sub-Continent. Nevertheless, the results for the Chinese women suggests the potential that GAIL can be improved for South-East Asian populations by using local (and/or updated) estimates of incidence and competing mortality rates.

Although the performance of GAIL-SBSP is in general satisfactory, at least amongst Singapore-Chinese women, one might anticipate that taking into account implications of the different health systems and etiological factors may produce improved prediction. For example, only 10% of the subjects in the SBSP cohort had had a mammogram in the previous year and this may have an impact on the apparent natural history of disease. More generally, women from much of the Asia-Pacific region do not receive regular mammogram screening [20]. Also, some factors included in GAIL may have different consequences in an Asian-Pacific population due to genetic predisposition, geographic, or other influences. To explore these aspects we initially used the same risk factors and coding that were in the original model of GAIL [1,2] to estimate relative risks with subjects from a case-control study, nested within the SBSP cohort (GAIL-SBSP (FULL)) and to compare these estimates with those from the BCDDP Table 2. Finally we derive a simplified model (S-GAIL-SBSP) with fewer risk factors and also an extended one (E-GAIL-SBSP) with additional risk factors included both incorporating local baseline race-specific breast cancer and other mortality rates.

S-GAIL-SBSP, which included age-at-menarche, age-at-birth of first live child and number of first-degree-relatives with breast cancer was well calibrated in the total SBSP cohort and across most subgroups (Tables 3 &4). It was not surprising that ‘ever having previous benign breast biopsy’ was not included in this revised prediction for Singaporean women as this reflects a specific health care delivery system in which biopsies were not common as is the case for the majority of Asian women (including Chinese-Occidental migrants) [20,21] although an increasing proportion of these women now receive mammographic screening [22,23]. Other evidence for the use of simpler, but more targeted, models has been provided by predictions of estrogen receptor-positive breast cancer in postmenopausal women in the USA [24]. However, the concordance from GAIL-SBSP (AUC = 0.61) is relatively low. This is similar to previous validation studies in non-Asian populations which have recorded an AUC between 0.56 and 0.60 for the GAIL model for Caucasian women [3], for the modified GAIL (CARE) an average age-specific AUC of 0.555 in African-American women [8], and 0.614 in Asian- and Pacific-Islander-American women [25]. Thus a good model with a higher discriminatory accuracy, in addition to good calibration, is needed [26]. Unfortunately, given the low relative risks associated with most established non-modifiable breast cancer risk factors, it is unlikely that any prediction model will have a much higher discriminatory accuracy [3,27].

The modest concordance suggests that additional factors prognostic for outcome may be required. In this respect we found that of bmi and parity were independent predictors of risk. Thus their inclusion in E-GAIL-SBSP, with modified *AR* and *RR*s derived from the SBSP cohort, marginally improved the discriminatory power (AUC = 0.62) but overestimated the predicted breast cancer cases substantially in, for example, the highest decile (Figure 1). One reason for this is that the *AR*s calculated may be inappropriate, possibly due to the true risk factor prevalence by age not taking a binary form with a cut at 50 years and/or influencing Asian women in different ways. Using the original *AR* values of GAIL made no improvement. Also overestimates may be a consequence of over-fitting a model with many risk factors based on only a modest number of cases and controls [28]. Similar mixed results were observed when the use of hormonal replacement therapy, oral contraception, smoking and waist-to-hip ratio were investigated (unreported analyses).

It has been suggested that the addition of mammographic breast density could provide improved discriminatory power for the GAIL model for Caucasian women [29,30] as the density is associated with an increased risk of breast cancer [13,31]. Further women with Tabar IV [32] parenchymal patterns amongst the SBSP cohort also have a significant higher risk of breast cancer when compared to those with the remaining patterns (odds ratio=2.30, CI=1.14 to 4.63). However those screened for this in the SBSP study were too few in number for us to validate any model that incorporates this risk factor.

The GAIL-SBSP, S-GAIL-SBSP and E-GAIL-SBSP models should always be applied with caution or avoided for certain specific populations as is true for GAIL itself. For example, although large SBSP was essentially confined (95%) to those of 50 and more years, they are applicable to younger women but further validation is needed. Further, we started the “clock” three years after negative screening which implies the SBSP based models are pertinent to women thought to be free of breast cancer. A woman who has just had a negative breast examination and mammogram, as the United Kingdom breast screening programme has shown, has about one third the absolute risk of breast cancer in the following three years [33]. Nevertheless, to establish the risks definitively, validation studies from a more representative sample of South-East Asian women in regular follow-up are required.

The strengths of this study include the use of a predominately postmenopausal group of women from three ethnic groups, Chinese, Malay and Indian, drawn from a very large screening program in which more than 29,000 women were randomly chosen to participate. Further, since neither Asian- or Chinese-Occidental (born in or migrated to the West) women were systematically included in the development of the Gail-based models [1,2]. We believe this is the first attempt to validate and modify the basic GAIL model to ethnically diverse women living in an Asian region. Earlier studies [21] have explored those of Chinese-Occidental origin who had migrated when aged less than 21 years (*N*=216) or had been residents in a western country for 10 years or less (*N*=421) [21] and Asian- and Pacific-Islander-American women [25].

Limitations of using SBSP data for individual absolute risk predictions include the inability of the nested case-control study to estimate elaborate models with sufficient precision. Also our validation data included relatively small numbers of breast cancer cases, especially amongst Singapore-Malay and -Indian women and those with two or more affected first-degree relatives. Furthermore, as with retrospective studies in general, the level of ascertainment of incident cases is of concern. However, Singapore is a small island where all citizens and permanent residents are registered in the population registry with a unique registration number. Also cancer notification is mandatory and this enables near complete ascertainment of breast cancer incidence by linkage of SBSP participants with the Singapore Cancer Register database.

## Conclusion

In conclusion, we found that among South-East Asian postmenopausal women, the GAIL type model could be refined using race-specific estimates of invasive breast cancer incidence and other cause mortality rates. A model which includes age-at-menarche, age-at-birth of first live child and number of first-degree-relatives with breast cancer appears to provide a simpler approach for projecting absolute risk of invasive breast cancer in South-East Asia women. Nevertheless its role in counseling the individual women regarding their risk of breast cancer remains problematical and needs to be validated in independent data.

## Competing interests

The authors declare that they have no competing interests.

## Authors’ contributions

FG, DM, SWD, DBM, KSC conceived the study and participated in its design. FG, DM, DBM, CHG, YFS participated in data analysis and interpretation. FG, DM, KYC, YFS, CHG drafted the manuscript. All authors read and approved the final manuscript.

## Pre-publication history

The pre-publication history for this paper can be accessed here:

http://www.biomedcentral.com/1471-2407/12/529/prepub

## References

[B1] Gail MH Brinton LA Byar DP Corle DK Green SB Schairer C Mulvihill JJ Projecting individualized probabilities of developing breast cancer for white females who are being examined annually J Natl Cancer Inst 1989 81 1879 1886 10.1093/jnci/81.24.1879 2593165

[B2] Costantino JP Gail MH Pee D Anderson S Redmond CK Benichou J Wieand HS Validation studies for models projecting the risk of invasive and total breast cancer incidence J Natl Cancer Inst 1999 91 1541 1548 10.1093/jnci/91.18.1541 10491430

[B3] Rockhill B Spiegelman D Byrne C Hnter DJ Colditz GA Validation of the Gail et al. Model of Breast Cancer Risk Prediction and Implications for Chemoprevention J Natl Cancer Inst 2001 93 358 366 10.1093/jnci/93.5.358 11238697

[B4] Spiegelman D Colditz GA Hunter D Hertzmark E Validation of the Gail et al. model for predicting individual breast cancer risk J Natl Cancer Inst 1994 86 600 607 10.1093/jnci/86.8.600 8145275

[B5] Kaur JS Roubidoux MA Sloan J Novotny P Can the Gail Model be useful in American Indian and Alaska Native populations Cancer 2004 100 906 912 10.1002/cncr.20047 14983484

[B6] Novotny J Pecen L Petruzelka L Petruzelka L Svobodnik A Dusek L Danes J Skovajsova M Breast cancer risk assessment in the Czech female population – an adjustment of the original Gail model Breast Cancer Res Treat 2006 95 29 35 10.1007/s10549-005-9027-5 16319995

[B7] Boyle P Mezzetti M Vecchia CL Franceschi S Decarli A Robertson C Contribution of three components to individual cancer risk predicting breast cancer risk in Italy Eur J Cancer Prev 2004 13 183 191 10.1097/01.cej.0000130014.83901.53 15167217

[B8] Gail MH Costantino JP Pee D Bondy M Newman L Selvan M Anderson GL Malone KE Marchbanks PA McCaskill-Stevens W Norman SA Simon MS Spirtas R Ursin G Bernstein L Projecting individualized absolute invasive breast cancer risk in African American women J Natl Cancer Inst 2007 99 1782 1792 10.1093/jnci/djm223 18042936

[B9] Seow A Koh WP Chia KS Shi LM Lee HP Shanmugaratnam K Trends In Cancer Incidence in Singapore 1968 – 2002: Singapore Cancer Registry Report No. 6 2004

[B10] Ng EH Ng FC Tan PH Ng EH Ng FC Tan PH Low SC Chiang G Tan KP Seow A Emmanuel S Tan CH Ho GH Ng LT Wilde CC Singapore Breast Cancer Screening Project Working Committee, Ministry of Health, Singapore Results of intermediate measures from a population-based, randomized trial of mammographic screening prevalence and detection of breast carcinoma among Asian women - The Singapore Breast Screening Project Cancer 1998 82 1521 1528 10.1002/(SICI)1097-0142(19980415)82:8<1521::AID-CNCR14>3.0.CO;2-6 9554530

[B11] Ng EH Gao F Ji CY Ho GH Soo KC Risk factors for breast carcinoma in Singaporean Chinese Women: the role of central obesity Cancer 1997 80 725 731 10.1002/(SICI)1097-0142(19970815)80:4<725::AID-CNCR11>3.0.CO;2-V 9264356

[B12] Gao F Chia KS Ng FC Ng EH Machin D Interval cancers following breast cancer screening in Singaporean women Int J Cancer 2002 101 475 479 10.1002/ijc.10636 12216077

[B13] Wong CS Lim GH Gao F Jakes RW Offman J Chia KS Duffy SW Mammographic density and its interaction with other breast cancer risk factors in an Asian population Br J Cancer 2011 104 871 4 10.1038/sj.bjc.6606085 21245860 PMC3048202

[B14] Singapore Department of Statistics Singapore Resident Population, 2003 – 2007 (February 2008) Available from http://www.singstat.gov.sg/pubn/popn/respop.pdf

[B15] Schonfeld SJ Pee D Greenlee RT Hartge P Lacey JV Jr Park Y Schatzkin A Visvanathan K Pfeiffer RM Effect of changing breast cancer incidence rates on the calibration of the Gail model J Clin Oncol 2010 28 2411 2417 10.1200/JCO.2009.25.2767 20368565 PMC2881722

[B16] WHO expert consultation Appropriate body-mass index for Asian populations and its implications for policy and intervention strategies Lancet 2004 363 9403 157 163 14726171 10.1016/S0140-6736(03)15268-3

[B17] Gail MH Pfeiffer RM On criteria for evaluating models of absolute risk Biostatistics 2005 6 227 239 10.1093/biostatistics/kxi005 15772102

[B18] Decarli A Calza S Masala G Specchia C Palli D Gail MH Gail Model for prediction of absolute risk of invasive breast cancer: independent evaluation in the Florence-European prospective investigation into cancer and nutrition cohort J Natl Cancer Inst 2006 98 1686 1693 10.1093/jnci/djj463 17148770

[B19] Chia KS Seow A Lee HP Shanmugaratnam K Cancer Incidence in Singapore 1993-1997. Singapore Cancer registry Report No. 5 2000

[B20] Kwong A Cheung PS Wong AY Hung GT Lo G Tsao M Chan EW Wong T Ma M The acceptance and feasibility of breast cancer screening in the East Breast 2008 17 42 50 Epub 2007 Aug 27 10.1016/j.breast.2007.06.005 17720500

[B21] Tam CY Martin LJ Hislop G Hanley AJ Minkin S Boyd NF Risk factors for breast cancer in postmenopausal Caucasian and Chinese-Canadian women Breast Cancer Res 2010 12 R2 Epub 2010 Jan 6 10.1186/bcr2465 20053286 PMC2880420

[B22] Yeoh KG Chew L Wang SC Cancer screening in Singapore, with particular reference to breast, cervical and colorectal cancer screening J Med Screen 2006 13 Suppl 1 S14 S19 17227636

[B23] Shin HR Joubert C Boniol M Hery C Ahn SH Won YJ Nishino Y Sobue T Chen CJ You SL Mirasol-Lumague MR Law SC Mang O Xiang YB Chia KS Rattanamongkolgul S Chen JG Curado MP Autier P Recent trends and patterns in breast cancer incidence among Eastern and Souteastern Asian women Canc Causes Contr 2010 21 1777 1785 10.1007/s10552-010-9604-8 20559704

[B24] Chlebowski RT Anderson GL Lane DS Aragaki AK Rohan T Yasmeen S Sarto G Rosenberg CA Hubbell FA Women's Health Initiative Investigators. Predicting risk of breast cancer in postmenopausal women by hormone receptor status J Natl Cancer Inst 2007 99 1695 1705 10.1093/jnci/djm224 18000216

[B25] Matsuno RK Costantino JP Ziegler RG Anderson GL Li H Pee D Gail MH Projecting Individualized Absolute Invasive Breast Cancer Risk in Asian and Pacific Islander American Women J Natl Cancer Inst 2011 103 951 961 10.1093/jnci/djr154 21562243 PMC3119648

[B26] Gail MH Personalized estimates of breast cancer risk in clinical practice and public health Stat Med 2011 30 1090 1104 10.1002/sim.4187 21337591 PMC3079423

[B27] Bondy ML Newman LA Assessing breast cancer risk: evolution of the Gail Model J Natl Cancer Inst 2006 98 1172 1173 10.1093/jnci/djj365 16954464

[B28] Steyerberg EW Vickers AJ Cook NR Gerds T Gonen M Obuchowski N Pencina MJ Kattan MW Assessing the performance of prediction models: a framework for traditional and novel measures Epidemiology 2010 21 128 138 10.1097/EDE.0b013e3181c30fb2 20010215 PMC3575184

[B29] Chen J Pee D Ayyagari R Graubard B Schairer C Byrne C Benichou J Gail MH Projecting absolute invasive breast cancer risk in white women with a model that includes mammographic density J Natl Cancer Inst 2006 98 1215 1226 10.1093/jnci/djj332 16954474

[B30] Barlow WE White E Ballard-Barbash R Vacek PM Titus-Ernstoff L Carney PA Tice JA Buist DS Geller BM Rosenberg R Yankaskas BC Kerlikowske K Prospective breast cancer risk prediction model for women undergoing screening mammography J Natl Cancer Inst 2006 98 1204 1214 10.1093/jnci/djj331 16954473

[B31] Boyd NF Guo H Martin LJ Sun L Stone J Fishell E Jong RA Hislop G Chiarelli A Minkin S Yaffe MJ Mammographic density and the risk and detection of breast cancer N Engl J Med 2007 356 227 236 10.1056/NEJMoa062790 17229950

[B32] Jakes RW Duffy SW Ng FC Gao F Ng EH Mammographic parenchymal patterns and risk of breast cancer at and after a prevalence screen in Singaporean women Int J Epidemiol 2000 29 11 19 10.1093/ije/29.1.11 10750598

[B33] Bennett RL Sellars SJ Moss SM Interval cancers in the NHS breast cancer screening programme in England, Wales and Northern Ireland Br J Cancer 2011 104 571 577 Epub 2011 Feb 1 10.1038/bjc.2011.3 21285989 PMC3049599

